# Response to “Creating Consensus: Revisiting the Emergency Medicine Scholarly Activity Requirement”

**DOI:** 10.5811/westjem.2019.1.42332

**Published:** 2019-02-14

**Authors:** Scott H. Pasichow, Zachary J. Jarou, Dhimitri A. Nikolla, Mohammed M. Qureshi, Michael L. Epter

**Affiliations:** * Brown University, Department of Emergency Medicine, Providence, Rhode Island; † University of Chicago, Department of Medicine, Section of Emergency Medicine, Chicago, Illinois; ‡ Saint Vincent Hospital, Allegheny Health Network, Department of Emergency Medicine, Erie, Pennsylvania; § Penn State Milton S. Hershey Medical Center, Department of Emergency Medicine, Hershey, Pennsylvania; ¶ University of Arizona College of Medicine, Creighton University School of Medicine, Department of Emergency Medicine, Pheonix, Arizona; || Maricopa Medical Center, Department of Emergency Medicine, Pheonix, Arizona

Dear WestJEM Editorial Board:

As representatives of the Emergency Medicine Residents’ Association (EMRA), the Council of Residency Directors in Emergency Medicine (CORD), the American College of Osteopathic Emergency Physicians - Residents and Student Organization (ACOEP-RSO), and the American Academy of Emergency Medicine - Residents and Students Association (AAEM-RSA) we write in response to “Creating Consensus: Revisiting the Emergency Medicine Scholarly Activity Requirement.”^1^ This paper presents the outcomes of efforts by the Society of Academic Emergency Medicine’s Research Directors Interest Group to understand emergency physicians’ attitudes and opinions on resident scholarly activity. We applaud the authors for their work on this challenging topic, and the editors for bringing it forward for discussion. However, we have some reservations about applications of its conclusions.

In emergency medicine (EM), our Accreditation Council on Graduate Medical Education (ACGME) Review Committee has granted wide latitude to programs when defining scholarly activity.^2^ A previous survey of EM programs found that a majority of program directors cited curriculum development, review articles, and lectures as ways in which residents adequately fulfill the scholarly activity mandate.^3^ Such activities were considered scholarly activity by the ACGME in the past,^2^ and maintained with the recent update to the Common Program Requirements, which were revised to mirror Boyer’s Model of Scholarship including “discovery, integration, application, and teaching.”^4^,^5^ The ACGME includes activities such as “grants,” “creation of curricula,” “electronic educational materials,” and “contribution to professional committees...or editorial boards”^4^ when defining faculty scholarly activity. These broad parameters encompass the spectrum of scholarship that exists in academic departments and embraces evolution, growth, and innovation in education. Kane et al. seeks to modify these requirement by suggesting that scholarly activity solely focus on the instruction of residents in scientific inquiry, and exposure to the mechanics of research. This change would narrow the definition of scholarly activity beyond what is currently accepted by the ACGME, and such an interpretation would preclude the use of national leadership and curriculum design for fulfillment of the scholarly activity requirement. While we appreciate the authors’ perspective, their scope of scholarly activity is of a more traditional research model and not of scholarship, which includes academic development and contributions. This would fall short of providing diverse opportunities to residents for how they use scholarly activity to grow their careers and our specialty.

Kane et al. made significant effort to have numerous opinions included in their consensus definition for scholarly activity. However, despite these efforts, CORD was absent from their in-person meeting. While CORD’s members responded to the survey, no subgroup analysis was performed, so viewpoints of the subset of emergency physicians who have the most direct contact with residents and their scholarly activity are not specifically outlined in this paper. This is a significant limitation to the consensus that these authors seek.

We also feel that the methodology used to interpret the survey fails to describe consensus. The cut point chosen to define consensus of 3.33 on a 4-point Likert scale makes it possible for 100% of respondents to “somewhat agree” with a statement and for this to not represent consensus. It also suggests that people who “somewhat agreed” with an option were actually voting against consensus on that item. The *American Journal of Public Health* recommends that, when building consensus, “if agreement of at least two thirds of participants can be reached...consensus is established.”^6^ This recommendation is more closely represented by a cut point of 2.66, which could have allowed case reports, curriculum design, or blog posts to count toward a consensus definition. Thus, the items included in their definition of consensus (and more importantly, those left out) cannot be meaningfully interpreted.

EMRA, CORD, ACOEP-RSO, and AAEM-RSA support a broad definition of scholarly activity that extends beyond the points proposed by Kane et al. We encourage the reader to consider the breadth of activity that contributes to the scholarly advancement our speciality when deciding what to require for trainees. There is real value in work which contributes to the discovery, integration, application, and teaching of emergency medicine, and we hope that the ACGME EM-RC will continue its practice of broadly defining scholarly activity, and not limit the future of this vibrant speciality.
