# Supplementary material for: Response to “Creating Consensus: Revisiting the Emergency Medicine Scholarly Activity Requirement”
Source: West J Emerg Med. 2019 Feb 14;20(2):376–9. doi: 10.5811/westjem.2019.1.42332 (PMC6404718; doi:10.5811/westjem.2019.1.42332)
Supplement: Related texts

# Reply: Response to Letter to the Editor

**Published:** 2019-02-14

**Authors:** Bryan G. Kane, Vicken Y. Totten, Chadd K. Kraus, Michael Allswede, Deborah B. Diercks, Nidhi Garg, Louis Ling, Eric N. McDonald, Alex M. Rosenau, Mike Wilk, Alexandria D. Holmes, Adam Hemminger, Marna Rayl Greenberg

**Affiliations:** * Lehigh Valley Hospital, Department of Emergency Medicine and Hospital Medicine, Allentown, Pennsylvania; † University of South Florida, Morsani College of Medicine, Lehigh Valley Campus, Allentown, Pennsylvania; ‡ Kaweah Delta Medical Center, Department of Emergency Medicine, Visalia, California; § Geisinger Health System, Department of Emergency Medicine, Danville, Pennsylvania; ¶ Mountainview Hospital, Department of Emergency Medicine, Las Vegas, Nevada; || University of Texas Southwestern Medical Center, Department of Emergency Medicine, Dallas, Texas; # Long Island Jewish Medical Center, Northwell Health, Department of Emergency Medicine, New Hyde Park, New York; ** Accreditation Council for Graduate Medical Education, Chicago, Illinois; †† University of Minnesota, Department of Emergency Medicine, Minneapolis, Minnesota; ‡‡ University of Mississippi, Department of Emergency Medicine, Oxford, Mississippi; §§ Brown University, Department of Emergency Medicine, Providence, Rhode Island

Dear *West*JEM Editorial Board:

We thank Pasichow et al. for taking the time to both read and comment on the consensus work reported in Kane et al.^1^ Foremost, our work was not intended to remove from individual program directors the ability to locally define scholarly activity. Program directors are already guided by a list of minimum expectations that the Review Committee for Emergency Medicine (EM) has labeled as “examples of acceptable resident scholarly activity.”^2^ Some programs will strive to achieve more than the minimum and prepare their residents for a higher level of scholarship and research. Pasichow et al.’s well-presented comments on the nature of scholarship are discussed in greater detail in an article published in the *West*JEMby Ander and Love.^3^ The article provides information on how to apply Boyer’s model, and provides both standards and a model to determine if a project meets a “test of scholarship.”

Our stated goal was to identify best practices for the scholarly requirement from as broad a perspective as possible. The original work dates back 20 years, and represents the specific views of research directors at that time.^4^ Medicine in general and EM in particular have evolved since then. Current Accreditation Council for Graduate Medical Education (ACGME) requirements include emerging emphasis on quality improvement.^5^ In response, our work has added quality improvement to a list of best practices for the scholarly requirement. Pressures from demographics and delivery of care continue to change the practice of EM.^6^ When combined with emerging technologies, our collective professional view of scholarship will also need to evolve. To address the influence of continued change in EM, there may be value in regularly revisiting the scholarly activity requirement on a more frequent basis. Both the upcoming changes to the ACGME Common Program requirements and their application by our Review Committee may impact when it is best to next revisit the scholarly requirement.^7^

In the end, stimulating dialogue such as that provided by this letter to the editor is the greatest opportunity for the application of our work. Hopefully, some of the resultant discussion will occur at the level of individual residency programs within the ACGME-required “Self Study” process.^8^ As each program sets its individual “program aims” and performs “strategic assessment” to “take the program to the next level,” our work and discussion such as this will hopefully be of value.

## References

[1] Kane B, Totten V, Kraus C (2019). Creating consensus: revisiting the emergency medicine resident scholarly activity requirement. West J Emerg Med.

[2] Accreditation Council for Graduate Medical Education Frequently Asked Questions: Emergency Medicine Review Committee for Emergency Medicine ACGME.

[3] Ander DS, Love JN (2017). The evolving definition of education scholarship: what the clinician educator needs to know. West J Emerg Med.

[4] Summers R, Fish S, Blanda M (1999). Assessment of the “scholarly project” requirement for emergency medicine residents: report of the SAEM Research Director’s Workshop. Acad Emerg Med.

[5] Accreditation Council for Graduate Medical Education ACGME Program Requirements for Graduate Medical Education in Emergency Medicine.

[6] Lin MP, Baker O, Richardson LD (2018). Trends in emergency department visits and admission rates among US acute care hospitals. JAMA Intern Med.

[7] Accreditation Council for Graduate Medical Education Common Program Requirements.

[8] Accreditation Council for Graduate Medical Education Self Study: eight steps for conducting the ACGME Program Self-Study.
